# Predictors of antiretroviral therapy interruptions and factors influencing return to care at the Nkolndongo Health District, Cameroon

**DOI:** 10.4314/ahs.v21i1.6S

**Published:** 2021-05

**Authors:** Marius Nsoh, Katayi E Tshimwanga, Busi A Ngum, Avelina Mgasa, Moses O Otieno, Bokwena Moali, Nathanael Sirili, Ndeso S Atanga, Gregory E Halle-Ekane

**Affiliations:** 1 Department of Public Health, School of Health Sciences, Catholic University of Central Africa; Cameroon; 2 HIV Free Project, Cameroon Baptist Convention Health Services, Center region; Cameroon; 3 Women Health Program, Mbingo Baptist Hospital, Cameroon Baptist Convention Health Services; Cameroon; 4 Ministry of Health Community Development, Gender, Elderly and Children; National Blood Transfusion Service; Tanzania; 5 National AIDS and Sexually Transmitted Infections Control Program (NASCOP); Kenya; 6 Ministry of Health and Wellness, Okavango District, Botswana; 7 Department of Development Studies, Muhimbili University of Health and Allied Sciences; Tanzania; 8 Department of Public Health, Obstetrics and Gynecology, Faculty of Health Sciences, University of Buea; Cameroon

**Keywords:** ART interruption, attrition, return to care, predictors, motivating factors, Cameroon

## Abstract

**Background:**

Antiretroviral therapy is a lifelong commitment that requires consistent intake of tablets to optimize health outcomes, attain and maintain viral suppression.

**Objective:**

We aimed to elicit predictors of treatment interruption amongst PLHIV and identify motivating factors influencing return to care.

**Method:**

We conducted a cross-sectional study using a mixed-method approach in four hospitals in Yaoundé. Sociodemographic and clinical data were collected from ART registers. Using purposeful sampling, thirteen participants were enrolled for interviews. Quantitative data were analyzed using Epi-Info and Atlas-TI for qualitative analysis. Ethical clearance approved by CBCHS-IRB.

**Results:**

A total of 271 participants records were assessed. The mean age was 33 years (SD±11years). Private facilities CASS and CMNB registered respectively 53 (19.6%) and 14 (5.2%) participants while CMA Nkomo and IPC had 114 (42.1%) and 90 (33.2%) participants. Most participants (75.3%) were females [OR 1.14; CI 0.78–1.66] compare with males. 78% had no viral load test results. Transport cost and stigmatization constituted the most prominent predictors of treatment interruption (47.5%) and (10.5%) respectively. Belief in the discovery of an eminent HIV cure and the desire to raise offspring motivated 30% and 61%, respectively to resume treatment.

**Conclusion:**

Structural barriers like exposed health facility, and dispensing ARVs in open spaces stigmatizes clients and increases odds of attrition. Attrition of patients on ART will be minimized through implementation of client centered approaches like multiplying proxy ART pick points, devolving stable clients to community ARV model.

## Introduction

Consistency on antiretroviral therapy (ART) remains the most effective intervention in the global HIV response and has proven an effective for people living with HIV (PLHIV). ART treatment interruption is a patient-initiated episode of more than 30 days of stopping ART but who will subsequently resumed treatment. When the rate of adherence to medication is as high as 95%, the viral suppression rate approaches 78%, however, when the rate of adherence is reduced to 80%, there is a dramatic reduction in the viral suppression rate, which can be as low as 20% thereby increasing odds of HIV transmissibility^1^. The adherence rate of medication should be maintained at 95% or above to optimize treatment outcomes and attain viral suppression. Since the discovery of ART in 1996, substantial improvement has been noticed on the path of HIV disease progression. According to UNAIDS, there were 38.0 million PLHIV worldwide by 2019. Global trends showed about 25.4 million were accessing antiretroviral therapy, up from 6.4 million in 2009 2. Incidence of HIV and its related deaths were 1.7 million and 770.000 respectively^3^. Attain and maintaining epidemic control requires acting on retention to achieve the best health outcomes associated with medication intake. Prior studies recommends an adherence of 95% or more to optimized health benefits associated with daily medication intake^4^. ART consistency maintain the virus in a latent state, and interrupt viral replication. Daily prescription for timely intake is important and psychosocial and counselling support provided by care providers to support consistency on ART. Monitoring and tracking of clients are required to provide support and adequately report to update the national health information system. By 2018 retention rate globally stood at 62% and moved to 73% by 2019 with significant variations per region, and Africa harboring 2/3 of the global burden^5^,^6^. ART being a lifelong commitment, requires consistency in treatment for all PLHIV to guarantee the continued effect of the medication, and reducing immune activation^7^. These drugs act within a time-lapse and, therefore, regular and timely intake is needed to maintain the virus in a latent state^8^. Viral suppression not only leads to improved clinical outcomes for the individual but also reduces the risk of drug resistance and HIV transmission^9^ to sexual or biologic contacts most at risk. This underscores the need to adhere diligently, follow the treatment schedule, take prescribed ART respecting appropriate time, doses, and frequencies^10^, to inhibits viral replication^11^.

In contrast, the absence of treatment, treatment inconsistency, and treatment interruption leads to quick viral rebound, replication, increased odds of drug resistance and increases the risk of opportunistic infections 12. Complete adherence to ART can prevent more than 96% of mother to child HIV transmission^13^,^14^ with concomitant decrease in morbidity and mortality 15. As interventions, establishing ART proxy pickup units on a peripheral level and task-shifting 16 contributes significantly to reduce problems associated with congestion in facilities, proximity barriers, and travel costs. However, stigmatization remains a challenge and contributes to attrition 17.

In the early years of the HIV/AIDS epidemic, the social consequences of stigma and discrimination towards people with HIV were identified as part of the “third phase of the epidemic” and addressing these consequences were “central to the global AIDS challenge as the disease itself ” to date stigmatization still prevent PLHIV from accessing treatment due to misconceptions by community and fear of critics 18. Cameroon harbored approximately 540,000 PLHIV by 2018, with 23,000 new infections and 18,000 related mortality to AIDS 19. According to UNAIDS by 2019, only 67% of all PLHIV on ART were retained on treatment showing a gap in retention. Some reasons explaining this gap include behaviors associated with stigmatization like traveling long distances for a refill^2^, ^20^. Weight gain after commencement of treatment to be a sign of cured 21,22. Other factors like, depreciating health state, unstable housing conditions, and frequent displacement increase the risk attrition rates 23. Intrinsic factors, such as sex and age, influence retention. Service delivery prone higher utilization for women and reduced health service uptake targeting men 24. Depression and other mental health problems are common comorbidities for PLHIV and are a cause of treatment interruption 22. On the other hand, health care providers and health system associated factors including poor patient-provider rapport 25, shortage of staff, inadequate space at ART clinics 21, concerns about confidentiality, inadequate counseling before initiation, and drug stockouts^26^ contribute to treatment interruption and attrition. Community-related factors like switched to traditional medicine^22^,^25^, the use of herbal preparations, fear of disclosure of status with partner, friends, siblings, and offspring^20^ are cited as negative influences on retention in care 21. Family pressures and religious beliefs also contribute to attrition. Stigmatization^25^ and discrimination in access to public services hinder adherence. In this context, ART treatment interruption is a patient-initiated episode of more than 30 days of stopping ART but who subsequently resumed treatment, while we defined retention in care as a patient who is still on ART (assessed at intervals longer than 12 months post-initiation) and has not died, transferred out, stopped treatment or been lost-to follow-up (LTFU). We aimed to identify predictors of TI amongst PLHIV on ART and understand motivating factors that influence return in care in selected hospitals in Yaoundé. This study arose in response to the growing rate of clients reported to have interrupted treatment and in remedy to understand reasons for this trends to guide policy makers in designing interventions aimed to raise retention and approaching epidemic control.

## Method

### Research design and approach Study design and setting

We conducted a mixed-method study using a cross-sectional design. This design enabled us to capture quantitative data, we later used purposeful sampling to select participants for in-depth interview from the quantitative population. The said approaches enabled us to explore the lived experience of participants and understand the meaning they attribute to the phenomenon of investigation included in the study were clients who initiated treatment in the four participating facilities from January 1^st^, 2016 to December 31^st^, 2018 but interrupted ART from September to December 2018, to enable us understand reasons associated with attrition, and generate evidence for policy guided interventions. Furthermore, in-depth interviews were also conducted.

### Study population

The study was conducted in the Nkolndongo health district, in Yaoundé. Participants were purposefully selected from four ART treatment units providing global HIV management to PLHIV. We included into the study all PLHIV who had interrupted treatment from September to December 2018 and returned by March 2019 and only those who did not give their consent to participate were excluded. To show variability the health facilities (HF) characteristics were disaggregated into private HF (CASS Nkolndongo and Medical Center Nicolas Barre (CMNB)) and public HF (CMA Nkomo and Infimerie prison Centrale (IPC)) settings. An exhaustive sampling targeting PLHIV 20 years and above was carried out. Subsequently, a consecutive sampling technic was used to enroll participants. in two different groups:

1. Early treatment interruption respondents (less than 12 months after ART initiation); and
2. Late treatment interruption respondents (more than 12 months after ART initiation).

### Sampling and sample size

No sample size was calculated for the quantitative assessment because we included all clients reported to have interrupted treatment during the investigation period. We purposefully sampled clients from the quantitative population for IDI and after 3 interviews per facility, we attained saturation of responses.

### Study procedures

We resorted to two of the most used data collection methods.

### Qualitative data

We conducted 13 interviews with non-structure probes to guide the respondents provide their lived experiences. We briefly voice a discussion topic then listen keenly to respond.

The interviews were recorded using an audio tape recorder, later transcribed, verbatim using Atlas-TI, and translated from French to English by the first author who is fluent in both languages and checked by other others. Related codes from themes were grouped and the responses categorized. Some topics covered included:

“What made you interrupt from care?”

“can you relate what you or other people think, prompt some people stick, stop or restart treatment?”

“From your experience or other people, you may know, relate how health services offered influence you/others decision to interrupt ART?”

“What personal/structural/community factors prompted you to return and stay in care?” and “In your opinion, what do you think motivated those who interrupted treatment to restart?”

### Quantitative data

We designed a data entry matrix using the software epi-info 7. We extracted demographic and clinical data on clients age, sex, date of initiation on ARV, duration on treatment, time of ARV interruption, and follow-up outcomes from registers and patient's files, follow up the patients through phone calls and if necessary, home visits to discuss their opinion on factors prompting TI and influencing factors to return in care. A comparable group of individuals who had not interrupted treatment with same characteristics, age, sex and cohorts was extracted to assess

### Outcome variable

Treatment interruption and resumption to care would be our outcome of interest.

### Data analysis

Quantitative data were entered and analyzed using EPI info 7.2. Simple frequency tables were made to view the data and perform cleansing prior to analysis. Univariate and bivariate analysis performed. Chi-square test performed to assess how significant the various factors influenced retention with the presentation of the p-value at 95% confidence interval . Viral load coverage was assessed with interest on viral suppression. A comparative group of clients who had not interrupted treatment with similar characteristics as time of initiation, and consistency on ARV was extracted to compare our variable of interest and assess measures of association.

### Qualitative data

Content data analysis was done with first step consisting of quality control of transcripts. All validated transcripts reviewed, and codes identified. Initial codes identified and generated based on predefined themes and codes informed by the interview guide. New codes were identified after review of transcripts. Both old and new codes classified under main and sub themes. The validated codes generated into Atlas-TI.

### Ethical considerations

Ethical clearance was obtained from the Cameroon Baptist Convention Health Services Institutional Review Board IRB (Ethics Clearance No. IRB2019-14). Written permission to start the study was also received from the District Medical Officer, Nkolndongo. All the ethical principles of informed consent, autonomy, and beneficence, as well as confidentiality, were observed.

**Table:** 

Description of codes used		
Variable	Categories	Description
Interview	IDI	In-depth interview
Sex	M	Male
	F	Female
Facility	IPC	Infimerie Prison Central
	CASS	Centre d'Animation Sociale et Sanitaire
	CMNB	Centre Medicale Nicolas Barre
	CMA Nkomo	Centre Medicale d'Arrondissement de Nkomo

## Results

There were 3342 patients on ART in the study sites. Two hundred and seventy-one missed an appointment and reported to have interrupted ART, table 1. The mean age of patients was 33 years (SD 11 years), 204 (75.3%) being female. The age group 25 – 49 years, registered the highest attrition, 208 patients (76.8%). Most, 192 (70.8%), were late treatment interruption. Public facilities had the highest treatment interruption rates: 90 (33.1%) and 114 (42.1%), for IPC and CMA Nkomo. After follow-up of those who had interrupted treatment, 95 (34.9%) on care, transport cost and distance were reported as major predictors 67 (47.5%) and 35 (24.8%) respectively. While stigmatization led to 28 (19.7%) of treatment interruption. Most of the participants, 212 (78.2%), had not attested for viral load since initiation. The details of the socio-demographic and clinical characteristics of participants are summarized in Table 2.

**Table 1:** Summary sociodemographic and clinical characteristics of participants per health facilities

Variable	Categories	CMNB Frequency (%)		CASS Frequency (%)		IPC Frequency (%)		CMA Nkomo Frequency (%)	
**Sex**	Male	5	36%	3	6%	35	39%	24	21%
	Female	9	64%	50	94%	55	61%	90	79%
**Age**	<24 years	2	22%	8	15%	2	4%	18	20%
	25 – 49 years	7	78%	39	74%	40	73%	68	76%
	>50 years	0	0%	3	6%	13	23%	4	4%
**Place of** **residence**	Catchment	11	79%	44	83%	69	77%	97	85%
	area within	3	21%	5	9%	7	8%	1	1%
	region out of region	0	0%	4	8%	14	16%	16	14%
**Duration to** **interruption**	Early TI	8	57%	38	72%	22	24%	34	30%
	Late TI	6	43%	15	28%	68	76%	80	70%
**Duration on** **ART**	<1 year	8	57%	15	28%	22	24%	34	30%
	1 – 2 years	6	43%	23	43%	41	46%	76	67%
	> 3 years	0	0%	15	28%	27	30%	4	4%
**Pre tracking** **outcome**	Missed appointment	6	43%	26	49%	39	43%	60	53%
	LTFU	8	57%	27	51%	51	57%	54	47%
**Tracking** **outcome**	On ART	9	64%	36	68%	66	73%	68	60%
	Not on ART	5	36%	15	28%	22	24%	34	30%
	Died	0	0%	2	4%	2	3%	12	11%
**n**			**14**		**53**		**90**		**114**
**Viral load**	Suppressed	8	73%	22	88%	19	83%	8	73%
	Unsuppressed	3	27%	3	12%	4	17%	3	27%
	**n**		**11**		**25**		**23**		**11**

**Table 2:** Distribution of participants according to socio-demographic and clinical characteristics

Variable	Modality	Frequency (N)	Percentage (%)
**Sex**	Male	67	24.7
	Female	204	75.3
**Age**	20 – 24 years	35	12.9
	25 – 49 years	208	76.8
	>50 years	28	10.3
**Interruption time**	Early TI	79	29.2
	Late TI	192	70.8
**Place of residence**	Yaoundé	221	81.5
	off Yaoundé	50	18.5
**Facility**	CASS Nkolndongo	53	19.6
	CMNB	14	5.2
	IPC	90	33.1
	CMA Nkomo	114	42.1
**Outcome by mid-2019**	Died	14	5.6
	Still not on treatment	86	31.7
	On treatment transfer out	76	29.9
	Restarted ART	95	34.9
**Reasons for interruption**	Transport cost	67	47.5
**of ART**	Had drugs	5	3.5
	Drug side effects	6	4.3
	Stigma	28	19.7
	Distance	35	24.8
**Viral load**	Suppressed	49	18.1
	Unsuppressed	10	3.7
	Never tested	212	78.2

### Association between socio-demographic characteristics and treatment interruption

Residing outside the center region increased by three-fold the risk of interrupting care. PLHIV on treatment for over one year were more likely to interrupt treatment as depicted in Table 3.

**Table 3:** Relation between socio-demographic characteristics and treatment interruption

Variables	Modality	TI n (%)	OT n (%)	OR [IC, 95%]	p_value
Age	20 – 24 years	35 (12.3)	32 (11.3)	I	I
	25 – 49 years	208 (73.2)	212 (74.6)	4.08 [0.45 – 36.78]	0.18
	>= 50 years	28 (9.9)	38 (13.4)	5.43 [0.58 – 51.25]	0.12

Sex	Female	204 (75.4)	207 (72.9)	I	
	Male	67 (24.6)	77 (27.1)	1.14 [0/78 – 1.66]	0.28

Area of residence	Around facility (Less than 5KM)	234 (82.4)	244 (85.9)	I	
	Within region (Less than 60Km)	37 (13)	36 (12.7)	0.93 [0.57 – 1.53]	0.44
	Outside region (More than 60KM)	13 (4.6)	4 (1.4)	0.30 [0.09 – 0.92]	0.02*

Duration on ART	Early Treatment Interruption	237(83.4)	202 (71.1)	I	
	Late Treatment Interruption	47 (16.5)	82 (28.9)	2.04 [136 – 3.06]	0.005*

### Description of qualitative participants

We interviewed all together 13 participants from the four participating facilities. 11 (84.6%) females and 2 (13.4%) males. 20% were aged below 24 years and 80% between 25 to 49 years. 4 interviews conducted in CASS, 4 IDI in IPC, 3 in CMNB and 2 in CMA

### Factors influencing treatment interruption based on qualitative analysis

#### Client-associated factors

Adequate patient counseling is important to ensure clients accept status and optimize adherence. The client needs to be psychologically ready. This is recommended to reduce potential attrition. Patients generally expressed readiness to initiate ART and stick on ART, especially pregnant women who knew it would prevent mother to child transmission. However, one said, “*Immediately I was told about my status, I thought of committing suicide and didn't listen to the care provider again. I just started treatment because the nurse told me to….*” (CASS_woman, 37 years old interrupted treatment 4 months and restarted).

Depression and negative mental state contributed to compromised treatment consistency. Clients generally expressed some form of psychological challenges that affected their decision to adhere to care. One patient stated, “*I had trauma when my sister's child living at my home got poisoned at the neighbor's house, this created a great psychological imbalance within me. added to my status this troubled me, I became depressed and lose concentration. I stopped ART for several months*” (CMNB_woman, 34 years old, interrupted treatment 8 months and restarted)

The desire to stop transmission to offspring was raised as a factor that encouraged clients who had interrupted treatment to restart ART. From the qualitative transcripts, client stated “*I have my other kid born HIV negative and it is because I was on treatment, this makes me restart treatment as I want my other kids negative and be strong and healthy to take care of them*” (CASS_woman, 24 years old, interrupted treatment 2 months and restarted)

#### Health system and care provider associated factors

Establishing trust and good care providers - client rapport, is recommended to optimize retention in care. care providers need to support the client and link them to the most convenient and closest facility to their residence. This reduces the financial burden breaks distance barrier and ensure smooth care providers-patients rapport. A patient stated, “*I was doing a little business that enabled me to pay the transport cost to the hospital. I relocated and later had to spend 10,000frs ($17) to come to the hospital from Nanga (more than 80 kilometers from Yaoundé) this made me interrupt treatment and I didn't want to get a transfer because I was not certain of care provider support in the new facility*.” (woman 30 years old interrupted treatment and restarted)

The attitude of care providers and organizational setting influenced the client's outcomes. Respondents expressed satisfaction regarding the type of care offered and the level of confidentiality yet, some patients experienced ill events and related: “*I was challenged with the fact the facility is too open and close to the roadside*” (IPC_man, 57 years interrupted treatment 2 months and restarted). “*Some health providers speak in quarters about client's status they recognize from the care unit. This morning, my neighbor a nurse was telling me about the status of a woman we know in the neighbor-hood this frustrated me, and I wondered if others don't speak about me too!*” (CMNB_woman, 34 years interrupted treatment 4 months and restarted).

“*I am sure they keep to our privacy and for this reason, I would not want to transfer to another location as I can't attest, I will get the same treatment that way*.” ( CASS_woman, 34 years interrupted treatment 2 months and restarted).

### Community-related factors and perceptions

The external influence of healers and spiritualists affects patterns concerning treatment outcomes. Most participants disclosed their status only to their partners or siblings; hence there was little influence from the community, but one related. “*Yes! once in the university teaching hospital (CHU), a lady I met there told me she had a product that could cure me. She told me to call hr, I believed her, I did, and she asked me to pay 600,000frs ($1000) for the first dose and later I can pay for the second after. She told me her treatment will cure me. This made me stop treatment hoping I will get this money and start her therapy but later did not have money*.” (CASS_woman, 34 years interrupted treatment 6 months and restarted).

### Motivation to return to ART

Disclosing the client's status to a partner or close relatives and friends created a free environment for the patient to intake daily doses comfortably as well as respect monthly ART pickup rendezvous. “*I share my status with my husband HIV who is HIV negative, he didn't reject me unlike others he supported me, he was somehow traumatized initially but now he accompanies me and reminds me of my daily medication times and pickup RDV*.” (CMA_woman, 33 years). “*I am a widow and my kids have been very supportive in reminding me of daily intake and monthly pickup. They provide me assistance that motivates me to adhere. My little daughter sometimes reminds me days before my visit to the facility*.” (CMNB_woman, 43 years)

The declining clinical condition of some clients and fear of dying persuaded them to return in care and restart treatment after interrupting and experiencing depreciating health states.

“*I returned to care when I was feeling too sick, consulted, and was advised to stay on the treatment I was made to understand that consistency on ART would increase my immunity and ability to fight opportunistic infections.*” (CMA_woman, 32 years).

Follow-up through phone calls and home visits encouraged clients to restart treatment. “*The frequent calls made, and home visit motivated me to retune to care and gain confidence once more*.” (CASS_woman, 33 years)

## Discussion

Retention remains a major challenge^29^ and stands as a major pillar in the global respond in the HIV/AIDS pandemic. In a study by Bulsara et al, demographic and economic factors increased odd of ART attrition^23^. These finding revealed a similar observation with economic factors influencing attrition patterns. Clients inability to afford transport cost to health facility were reluctant to pick monthly ART doses, as a result, they interrupted care. Similar findings were seen in the qualitative analysis were PLHIV reported to had interrupted care as they could not afford transport cost to the ART pick up health facility. The predominance of women 75.3% compared with men 24.6% observed was due to the feminization of health services like antenatal clinic and low male uptake of health services. Similar trends were observed in Ethiopia whereby 64% female compared with 34% male experienced interruption^20^,^25^ this disparity was observed as women utilize health services most with more service packages targeting them in contrast with men who showed reduced utilization of health services. Clients in private HF experienced more support and follow-up coupled with the accommodating environment^30^. They had more devoted staff, who worked extended hours. This was different in the public facilities where clients were received during limited hours contributing to increase attrition. Mukumbang et al, also observed private facilities offered a better infrastructure and environment promoting privacy, safety, security, and confidentiality compared with public facilities^20^, These correlated with qualitative findings where clients affirmed comfort, confidentiality and hesitated to be transferred hence interrupted treatment when she could not travel to the treatment center. Infirmerie Prison Central had a high population of prisoners who interrupted care shortly after release from prison. This was due to inadequate counseling before their release equally observed. Form the patient's perspective, displacement, and travel constituted the major reasons for TI. Moreover, residents out of Yaoundé presented twice the risk of TI compared with those on treatment OR 0.30 CI;[0.09–0.92) P=0.02. This is an evidence of stigmatization as clients feel reluctant to pick up salvage dispensation or transferred to nearby facilities. This was similar to Lifson et al. findings that identified travels and stigma in raising attrition^27^–^29^. The mental state of PLHIV and their relationship with care providers affected their consistency in care. A thorough client assessment is required before ART initiation. Adequate counseling and psycho-emotional support are recommended to reduce the feeling of rejection and depression^30^,^31^ while reducing misconceptions about HIV. Persistent psychosocial and emotional support from care providers, phone calls, and home visits were cited as major interventions that strengthened client-care provider reports. Bulsara et al. findings revealed subjects with poor mental state showed a greater risk of attrition^24^. Most interview responded reported attrition when depressed and stressed psychologically. In the qualitative analysis, client reported support from care provider and frequent call greatly strengthened client-care providers rapport and this influenced their will to stick on ART and motivated resumption on care and treatment. The sharing of HIV status enabled PLHIV to gain support from partners or children. The latter reminded them of daily doses intake and monthly pickup appointments. Similar observations made by Alan et al. were disclosure of status improved social support^28^ and better retention. Drug side effects influence the client's decision to interrupt treatment. About 5% of clients interrupted treatment due to adverse drugs effects. Clients in qualitative assessment also revealed drugs effects prompted them to interrupt treatment though they used fruits like “pineapple, oranges”, to attenuate these effects. Other studies mentioned these effects as compromising the quality of life.

Clients believe in a forthcoming discovery of an HIV cure^32^,^33^, stories from the patients cured using stem cell transplants raised hope in PLHIV especially expressed in the qualitative assessment and influenced positively retention in care where participants revealed to be in expectation of a future cure reason they should stick on treatment. Follow-up through phone calls, home visits strengthened the clients-care provider relationship leading to better retention similar observation made by Nathalie et al 34. Vrazo, also observed home visits improved retention in care 35. We likewise observed women generally expressed relief after having an HIV negative outcome for their babies. This motivated them to adhere to their ART. The desire to raise offspring, and the support received from family, influenced PLHIV decision to restart and continue ART. In the qualitative interview's female participants reported to restart ART as they wanted to prevent transmission to their offspring's. Cecilia, et al findings revealed social support contributed to promoting treatment resumption^36^. Early active follow-up of patients improved retention in treatment. Our observation also revealed the need to track clients soon as they default to improve retention. Clients also related belonging to a support group and learning and economic activity might reduce their financial burden hence enabling them to assume related charges to attain monthly visits.

## Limitations

-Double coding for qualitative analysis not done.

-Few health facilities enrolled, and hence bigger studies recommended.

## Recommendations

-Similar study be conducted on a national scale for more robust conclusions and improve patients care and treatment.

-Keen attention and clinical follow up be made for clients who interrupt treatment especially viral load monitoring.

## Conclusion

Social, economic, and health system factors influence a client's therapeutic outcomes and retention in care. This study also revealed that client's inability to bear transport cost to the health facility and unreadiness to be transfer to a proxy treatment center to their homes influenced retention in care. Prominent factors influencing retention in care included stigma, drugs side effects and care provider patient rapport. Therefore, ART attrition will be minimized through implementation of strategies that further reduce these structural, and socioeconomic barriers. This stresses the need to reinforce psychotherapy throughout treatment cascade and educating health care providers on patient-centered approaches to optimize retention.
